# The Sponge Hologenome

**DOI:** 10.1128/mBio.00135-16

**Published:** 2016-04-21

**Authors:** Nicole S. Webster, Torsten Thomas

**Affiliations:** a Australian Institute of Marine Science, Townsville, QLD, Australia; b Centre for Marine Bio-Innovation, University of New South Wales, Sydney, Australia

## Abstract

A paradigm shift has recently transformed the field of biological science; molecular advances have revealed how fundamentally important microorganisms are to many aspects of a host’s phenotype and evolution. In the process, an era of “holobiont” research has emerged to investigate the intricate network of interactions between a host and its symbiotic microbial consortia. Marine sponges are early-diverging metazoa known for hosting dense, specific, and often highly diverse microbial communities. Here we synthesize current thoughts about the environmental and evolutionary forces that influence the diversity, specificity, and distribution of microbial symbionts within the sponge holobiont, explore the physiological pathways that contribute to holobiont function, and describe the molecular mechanisms that underpin the establishment and maintenance of these symbiotic partnerships. The collective genomes of the sponge holobiont form the sponge hologenome, and we highlight how the forces that define a sponge’s phenotype in fact act on the genomic interplay between the different components of the holobiont.

## DEFINING THE SPONGE HOLOGENOME

Sponges (phylum Porifera) are among the most ancient of the extant multicellular organisms, evolving over 580 million years ago and encompassing over 8,600 formally described and 15,000 estimated species that are distributed across shallow and deepwater habitats from the tropics to the poles (1). Sponges are ecologically important constituents of benthic environments, as they can occupy up to 80% of the available substrate, provide habitat for a wide range of infauna species, couple the benthic and pelagic zones through their filtration of enormous quantities of seawater, and mediate biogeochemical fluxes by respiring organic matter and facilitating the consumption and release of nutrients, including nitrate, nitrite, ammonium, and phosphate (2–5). Sponges metabolize a significant component of a reef’s primary production and can also recycle organic carbon through shedding of cellular material, which is rapidly consumed by detritivores (6).

Despite their simplistic body plan, which places cells in direct contact with the surrounding seawater, sponges are known for hosting dense, diverse, and highly specific microbial communities (7). These microbial symbionts can comprise up to 35% of the host’s biomass and make valuable contributions to many aspects of the sponge’s physiology and ecology. For this reason, sponges are described as “holobionts,” that is, a unit comprised of the sponge host and the consortium of bacteria, archaea, unicellular algae, fungi, and viruses that reside within it (8). It should be noted that the roles of sponge-associated fungi, viruses, and unicellular algae have been little studied compared to those of other members of the sponge holobiont.

Sponges and/or their associated microorganisms produce a wide range of secondary metabolites known to provide protection against predators and epibionts, and this chemical defense is considered a major contributor to the evolutionary and ecological success of sponges (reviewed in reference 9). Recent genetic and genomic investigations of the biosynthetic pathways for these compounds have generated valuable insights into the origin (i.e., sponge or symbiont derivation) of the sponge’s natural product chemistry and, in some instances, also revealed the genes necessary for heterologous biotechnological production of the compounds (10, 11). While studies are showing that bacterial symbionts are of central importance in the chemistry of sponges, the complexity and diversity of homologous genes associated with sponges of biomedical interest still represent a major challenge for identification of specific biosynthetic genes.

While sponge symbiosis has been the focus of numerous targeted reviews (7–9, 12–14), here we further state that the collective genomes of the sponge holobiont form the “sponge hologenome.” The review begins by describing the diversity, specificity, and functions of sponge microbial symbioses before exploring transmission mechanisms and recent findings about the physiological and molecular interactions that underpin the stability and maintenance of the sponge holobiont. We finish by discussing the adaptation of the holobiont to changing environmental conditions, including processes that may facilitate hologenome evolution, as well as by identifying emerging research priorities for the field. The priority research areas that are emerging as frontiers for the field of sponge microbiology include the following.

1. **Defining the molecular determinants of sponge holobiont interactions.** Insights into the molecular mechanisms mediating sponge-microbial interactions can be achieved by single-cell and (meta)genomic/transcriptomic sequencing efforts, as well as molecular, biochemical, and physiological studies.
2. **Defining spatial differentiation of function.** The extent to which sponge microenvironments (e.g., pH, redox potential, and oxygen concentration) vary across the sponge habitus and how this influences holobiont function should be investigated.
3. **Defining the extent of coevolution in sponge holobionts.** The extent of coevolution within the sponge hologenome and whether sponge microbiomes have undergone natural selection that benefits the host should be ascertained.
4. **Combining molecular sequence data with experimental research.** To reveal unique insights into the molecular pathways employed by both host and symbionts, it is essential for the field of sponge hologenomics to move from describing “functional potential” to hypothesis-driven, experimental research that can confirm putative symbiont physiologies and determine shared metabolic pathways.
5. **Defining how host innate immunity mediates the sponge microbiome.** Our understanding of the interplay between host immunity and the microbiome in maintaining the sponge holobiont should be expanded.
6. **Evaluating the extent to which sponge microbiomes enhance environmental adaptation of the holobiont.** Whether environmentally induced fluctuations in the sponge microbiome can have significant functional consequences for the holobiont phenotype upon which selection may act should be ascertained.
7. **Determining the extent of functional equivalence and functional redundancy in sponge microbiomes.** The extent to which functional redundancy within a microbiome influences holobiont stability and vulnerability to environmental change should be investigated.

## SPONGE MICROBIAL ASSOCIATIONS: ABUNDANCE, DIVERSITY, AND SPECIFICITY

**FIG 1:**
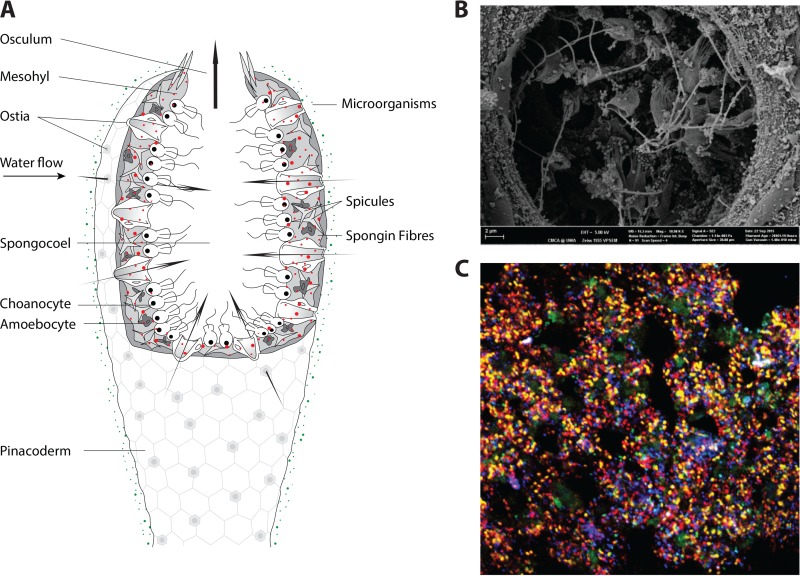
(A) Overview of a marine sponge body plan showing a schematic representation of an asconoid sponge. For the more common leuconoid sponge morphology, see the detailed schematic representation in reference 9. Seawater enters the sponge through tiny pores (ostia) in the surface layer (pinacoderm). Flagellated choanocyte cells are arranged in rings lining the sponge’s aquiferous system and are responsible for moving water through the sponge until it is discharged via the exhalant opening (osculum). Environmental microorganisms are shown as green cells, whereas the dense community of symbiotic microorganisms that exists within the mesohyl is portrayed as red cells. Siliceous spicules provide additional structural support to many sponge species. (B) Multicolor double labeling of oligonucleotide probes for fluorescence *in situ* hybridization (DOPE-FISH) analysis of the sponge mesohyl using rRNA-targeted probes for specific sponge-associated microorganisms reveals the density of microbial cells. *Poribacteria*, yellow; *Nitrospira*, pink; *Chloroflexi*, cyan; *Deltaproteobacteria*, light green; *Gammaproteobacteria*, red; *Archaea*, blue. Larger dark-green areas are sponge autofluorescence. Photo courtesy of Michael Wagner, Department of Microbial Ecology, University of Vienna. (C) Scanning electron micrograph showing the density of microbial cells surrounding the sponge choanocyte chamber, where individual flagellated choanocyte cells are responsible for drawing water through the sponge and removing bacteria and other particles for subsequent digestion. Photo courtesy of Peta Clode, Centre for Microscopy, Characterisation and Analysis, University of Western Australia.

Sponge-associated microorganisms generally occupy the sponge extracellular matrix (mesohyl), where they are concentrated around the choanocyte chambers, rings of flagellated cells that form the basis of the sponge’s aquiferous system (Fig. 1). However, some sponge species also host endosymbionts within specialized bacteriocyte cells (reviewed in reference 7), and high densities of cyanobacteria or microalgae are also often detected just below the pinacoderm cell layer, where they are maximally exposed to sunlight, which is required for phototrophy (15). While sponges can host enormous densities of microbial symbionts, reaching 10^9^ cells/cm^3^ of sponge tissue, it is important to note that not all sponge species harbor such a high microbial abundance (HMA). Some species are considered low-microbial-abundance (LMA) sponges, as they host only 10^5^ to 10^6^ bacteria/cm^3^ of tissue and maintain mesohyl regions with few microorganisms (16, 17). Quantitative data on the abundances of specific taxa within sponge tissue remain scarce, although recent studies employing quantitative PCR (qPCR) of select sponge species have determined the abundances of bacteria, archaea, chloroflexi, actinobacteria, cyanobacteria, and poribacteria and discerned notable differences that relate to the overall microbial abundance within the host (18–22). While this HMA-LMA dichotomy is a topic of current research, little is yet known about why particular sponge species host so few symbionts and whether the interactions within the hologenomes of LMA species are fundamentally different from those of their HMA counterparts. However, metagenomic profiling of microbiomes of HMA and LMA sponges has shown that at least some functional features are shared between these two groups (23, 24).

In contrast to humans, whose microbiome is characterized by high species diversity but low phylum-level diversity (25), sponges are known to associate with microorganisms spanning a remarkable 52 different microbial phyla and candidate phyla (26–28). The levels of richness and diversity of these symbiont communities vary widely between sponge species, ranging from a few distinct operational taxonomic units (OTU) to thousands of genetically distinct symbionts per host taxon (26, 28, 29), most of which are considered metabolically active (30). Extensive phylogenetic surveys have revealed that the dominant sponge-associated microorganisms reside within the taxa *Gamma*- and *Alphaproteobacteria*, *Actinobacteria*, *Chloroflexi*, *Nitrospirae*, *Cyanobacteria*, the candidate phylum “*Poribacteria*,” and *Thaumarchaea* (9, 31).

A considerable body of research has shown that sponge microbial communities are largely specific to host species (26, 27, 29, 32, 33) and that within a species, microbial communities tend to be highly stable across biogeographies and different environmental conditions (8, 28, 34). Some of the associated microorganisms are also shared between different sponge species, with phylogenetically unrelated sponges from geographically distant oceans sharing microbial phylotypes that are either still undetected or very rare in any nonsponge environment (7, 35, 36). This remarkable host phylum specificity initially led Hentschel and colleagues to introduce the term “sponge-specific 16S rRNA gene sequence clusters” (SC), which were defined as groups of sequences that were both phylogenetically more similar to each other than to sequences from non-sponge sources and also derived from two or more sponge species or the same species from different geographic locations (35). An example of this is a particularly large SC for the cyanobacterium “*Synechococcus spongiarum*,” which contains 245 unique sequences derived from over 40 different sponge species (31). Phylogenetic surveys undertaken over the last decade have determined that around 30% of all sponge-derived microbial sequences can be assigned to SCs, with the degrees of sponge specificity varying across taxa and ranging from very few SCs within the *Bacteroidetes* to a large number in the candidate phylum “*Poribacteria*” (31). However, recent deep surveys of 16S rRNA gene diversity have revealed that some of these SCs also occur at extremely low abundances outside sponges, suggesting that the terminology describing these clusters should be revised to “sponge-enriched SC” (i.e., SESC) rather than “sponge-specific SC” (18, 37).

The application of host-symbiont network analysis to 16S rRNA gene surveys can provide a valuable approach for exploring ecological and evolutionary dynamics within holobionts. These tools have only recently been applied to sponge holobionts and are generating interesting insights into the complex web of interactions and species distributions that can occur in sponges (38, 39) as well as in other symbiotic systems (40). For instance, in ecological systems, both theoretical and empirical studies have shown that communities skewed toward many weak and few strong interactions can enhance population stability and generally arise during the assembly of persistent communities (41, 42). Similarly, mutualism or skewed interactions affecting only one interacting partner, such as would occur in amensalism or commensalism, have been shown to promote diversity and lead to community stability (43, 44). Unique insights into sponge-symbiont interactions and distributions are expected to be revealed by the utilization of such networking tools.

## PROCESSES OF SYMBIONT TRANSMISSION AND ACQUISITION

Sponges can reproduce both sexually and asexually, with the latter occurring via fragmentation, budding, or production of gemmules, all of which can effectively transfer the symbionts of the parent sponge. In terms of sexual reproduction, sponges can be hermaphroditic (contain both male and female reproductive parts in a single individual) or gonochoristic (contain either male or female reproductive parts in a single individual) and exhibit both viviparous (larvae are developed within the sponge body) and oviparous (gametes are expelled for external fertilization outside the sponge body) development (45). For maintenance of the sponge hologenome, inheritance of symbionts needs to occur either directly through parental gametes (i.e., vertical transmission), through faithful acquisition from the surrounding environment (i.e., horizontal transmission), or through a mixture of both strategies (46). Each of these strategies has certain benefits and costs (reviewed in reference 13). For instance, while a strategy of vertical transmission ensures that offspring obtain the symbionts that are necessary for host fitness directly from the parent (47), it can also result in reduction of the symbiont genome or loss of metabolic capability over successive generations (48), and this may be deleterious in cases where larval dispersal results in sponge distributions to habitats that are suboptimal for the symbionts (13). While natural selection may therefore seemingly favor hosts that acquire their symbionts from the local environment (47), horizontal acquisition has its own risks for maintenance of the holobiont, including the availability of the symbionts within the surrounding environment during key acquisition times and the possibility that “cheater” symbionts or putative pathogens can exploit the host acquisition mechanisms to coinfect the sponge host (13, 49). A so-called “leaky vertical transmission” strategy (47) may balance the respective pro’s and con’s of vertical versus horizontal symbiont transmission, although the extent to which this occurs in sponge symbiosis still remains to be determined. The majority of research into sponge symbiont transmission has focused on viviparous species, describing the microbiome that is shared between adults and the developing embryos or fully developed larvae. Vertical transmission of highly diverse microbial assemblages comprising many (or most) of the taxa present in the adult microbiome has been demonstrated for numerous sponge species (26, 50–52), although there is still little knowledge of how stringent this vertical transmission process is (i.e., whether the symbionts also occur in the rare biosphere of the seawater) and whether these populations persist or change during larval metamorphosis and subsequent juvenile development. While vertical transmission of symbionts via the male germ line is considered extremely rare in animals due to the inherent streamlined sperm structure (53), a remarkable exception is known with the transmission of cyanobacterial symbionts in sperm of the sponge *Chondrilla australiensis* (54).

An enduring question in the field of sponge microbiology was how distantly related species from geographically isolated regions managed to acquire shared microbiomes when the symbionts were seemingly absent from the surrounding environment. As mentioned above, the application of deep sequencing has revealed that many sponge symbionts may indeed exist in low abundance in seawater, and hence the environment may act as a reservoir to populate phylogenetically distinct or geographically distant sponge individuals. While this seeming incongruence between highly specific symbioses and global symbiont distribution can be explained by a model of combined vertical and horizontal symbiont transmission, it is still unclear whether coinheritance occurs to the degree that evolution actually acts upon the hologenome interactions. We also have little understanding of the symbiont’s contribution to establishing and maintaining these associations. For example, it has recently been shown that coral-associated microorganisms display high levels of chemotaxis to chemicals released by the coral holobiont (55, 56), and it is feasible that sponge symbionts may employ similar mechanisms to actively seek out their sponge hosts.

The significance of horizontal versus vertical transmission mechanisms for maintaining sponge hologenomes can also be assessed by considering the patterns of symbiont sharing and the degree to which closely related sponge hosts share symbiont communities (codivergence or phylosymbiosis). For instance, if sponges relied predominantly on horizontal transmission of symbionts, one would not expect the microbial community to change in parallel with host nuclear phylogeny, unless of course the interactions within the hologenome generated specificity and codivergence. Community dissimilarity has been used in recent 16S rRNA gene surveys to detect some evidence of a phylogenetic signal in sponge microbiomes (57, 58), although other studies have found little similarity between closely related host species (27, 59). There is also little evidence to support the notion that microbial diversity in symbiont communities gradually increases with evolutionary time (57). Interestingly however, sponge species with similar eco-evolutionary characteristics—defined as the filtration rate, transmission strategy, and proportion of tissue occupied by microbes—were recently found to have similar phylogenetic bacterial community structures and temporally persistent host-bacterium associations, whereas other sponge species exhibited microbial turnover more consistent with that of planktonic microbiomes (60). At this point, it is also important to highlight the idea that observing greater community similarity among more closely related hosts does not necessarily imply that symbionts have coevolved with their hosts (61). Additional work is required to fully explore the extent of coevolution in sponge holobionts and determine the forces that drive the development and transmission of high symbiont complexity in these early-diverging metazoans.

## PHYSIOLOGICAL INTERACTIONS WITHIN THE SPONGE HOLOGENOME

While there is considerable knowledge about the diversity and specificity of microbial associations in sponges, little is in fact known about the physiological interactions that occur within the holobiont. Sponge symbionts are presumed to benefit from a stable supply of nutrients and access to ammonia, which is excreted from the host, while the host is presumed to benefit from supplemental nutrition or waste removal derived via a diverse range of metabolic processes provided by the symbiont community. Although the proposed symbiotic functions are extensive (reviewed in references 7 and 9), examples of specific symbionts being unequivocally assigned functional roles are actually quite rare. Abundant sponge symbionts are so far recalcitrant to cultivation, and this and the lack of a tractable cell–biological-host model or axenic cell cultures for experimental manipulation are major constraints to unequivocally linking symbiont identity and function. Despite these limitations, single-cell and metagenomic sequencing approaches combined with physiological experiments are starting to provide insight into the metabolic pathways employed by sponge symbionts, including carbon and nitrogen metabolism, autotrophic sulfur oxidation, and vitamin synthesis as well as a diverse array of transporters indicating extensive metabolite import into the symbionts (9, 24).

### Carbon.

With respect to carbon metabolism, many tropical sponge species harbor dense populations of photosynthetic cyanobacteria, and translocation of photosynthates from the symbiont to the host (mostly in the form of glycerol) has now been demonstrated for several species (reviewed in reference 7), with some sponges obtaining greater than 50% of their energy requirements from symbiotic cyanobacteria (62). However, the abundance of cyanobacteria in these sponge species has raised questions about how the sponge holobiont controls the size of the symbiont population so that the host tissues are not completely overwhelmed. Host consumption of excess symbionts, forced symbiont expulsion, and stealing of photosynthates by the host have been proposed to limit the symbionts (63), but as yet no evidence has been provided to support any of these scenarios. The molecular mechanisms involved in moving from a state of symbiont maintenance under low population densities to recognition as food under high population densities is seemingly extremely complex.

A number of recent stable-isotope studies have been conducted to quantify nutrient assimilation by symbionts as well as the transfer of symbiont-derived carbon and nitrogen to the sponge host (64–67). Overall, these studies demonstrated that transfers of carbon from symbiont to host differ substantially among different sponge species (64, 66, 67) and that the primary determinants of nutrient transfer are host-symbiont identity, irradiance, and the ratio of productivity to respiration (66). Most recent experimental analyses with closely related *Aplysina* species revealed a tight coupling of host and symbiont metabolism in *Aplysina cauliformis* but a weak coupling in *Aplysina fulva*, further emphasizing the species specificity of sponge microbiomes and suggesting that even closely related holobiont species may be on distinct evolutionary trajectories (67).

Genomic sequence analysis has also provided recent insight into the pathways for carbon metabolism in sponge holobionts, revealing genes encoding proteins involved in the glycolysis and pentose phosphate pathways, tricarboxylic acid cycle, 3-hydroxypropionate cycle, and oxidative phosphorylation (68, 69). In addition, the first complete genome of the cyanobacterium “*Candidatus* Synechococcus spongiarum,” which occurs in many different sponge species from multiple geographic locations, encodes low-molecular-weight peptides of photosystem II (*psb*), enabling low-light photosynthesis, but lacks a gene for methionine (70), indicating genome streamlining as an adaptation to the sponge’s intercellular environment. While it is suspected that many sponge species are mixotrophs (derive nutrition from both autotrophy and heterotrophy), the nutritional interactions and reciprocal feeding patterns between the different components of sponge holobionts are rarely explored and are still largely unknown.

### Nitrogen.

Nitrogen metabolism and cycling have been extensively studied in microbial communities of sponges. Ammonia oxidation is perhaps the best-studied symbiotic function in sponge holobionts, with a wide diversity of both ammonia-oxidizing archaea and bacteria being known to inhabit a large diversity of sponges (24, 71–75) and with at least some of these being vertically transmitted (76). Gene-centric (i.e., *amoA* PCR), genomic, metagenomic, metatranscriptomic, metaproteomic, and other experimental studies have revealed the importance of symbiont-driven ammonia oxidation in the sponge holobiont (24, 72–78). Genes involved in the initial steps of denitrification (i.e., nitrate and nitrite reduction) are also enriched in symbiont genomes from many sponge species (24, 69, 79); however, few genes for nitric oxide and nitrous oxide reductases, which are required for the subsequent steps of denitrification, have been detected. This led Fan and colleagues to postulate that incomplete or alternative pathways for denitrification may occur in the holobiont (24). Limited oxygen diffusion (e.g., caused by ceased pumping) can rapidly create anoxic conditions in sponge tissue, thus facilitating anaerobic denitrification (80). Recent evidence has shown that nitric oxide produced by incomplete denitrification can be microbially converted to nitrogen and oxygen (81), and this may partially restore aerobic conditions and respiration, although targeted experiments are required to support such a process in sponges. In addition, urease activity has been identified in the sponge *Xestospongia testudinaria* (82), and urease-encoding gene clusters and urea transporters are also reported from sponge metagenomes (69, 83). Urea is one of the dominant organic nitrogenous compounds in oligotrophic oceans (84) and likely serves as an alternative nitrogen source to ammonia, nitrate, and nitrite within the sponge holobiont.

### Sulfur.

Both sulfate-reducing and sulfur-oxidizing microorganisms inhabit sponges, with sulfur-oxidizing bacteria (SOB) thought to oxidize the reduced sulfur compounds generated by sulfate-reducing bacteria (SRB). To understand the role of sulfur metabolism within the sponge holobiont, Hoffmann and colleagues performed *in situ* isotopic measurements using *Geodia barretti* and found exceptionally high sulfate reduction rates (85). Subsequent lipid biomarker analysis indicated that SRB-derived carboxylic acids were likely transferred to the host, and SOB activities combined with chemical reoxidation processes were thought to prevent a toxic buildup of sulfide. However, additional studies are still required to determine whether the anaerobiosis reported for *G. barretti* occurs in other sponge holobiont models. Recently, the first genome of a sponge-associated SOB revealed a highly versatile capacity for carbohydrate uptake as well as autotrophic and heterotrophic metabolism that might help to maintain the holobiont association under a variable supply of reduced sulfur compounds (86).

### Phosphate.

A recently identified symbiont function spanning three phylogenetically disparate sponge species is microbial production and storage of polyphosphate granules, which can comprise 25 to 40% of total phosphate present in sponge tissue (5). This finding not only has major consequences for our understanding of nutrient cycling on coral reefs but also raises questions about the selection pressure that has resulted in the evolution of this particular symbiont function. Phosphate is important for regulatory functions within the holobiont (87), and it is conceivable that polyphosphate granules serve as a means of energy storage, as they are formed by phosphoanhydride bonds, which are analogous to those in ATP. Colman (88) proposes that these polyphosphate granules may actually be used to protect the holobiont against periods of phosphate deprivation, which, if validated, would be one of the first examples of microbial endosymbionts storing nutrients and energy for their host and would represent another unique mechanism that could sustain the sponge holobiont.

### Vitamins.

The biosynthesis of essential vitamins has also been reported as a key symbiont function in sponge holobionts, with considerable potential benefits for the host animal. Genomic and metagenomic studies have revealed that sponge symbionts are enriched in genes involved in the synthesis of vitamins, including vitamin B_1_ (thiamine), vitamin B_2_ (riboflavin), vitamin B_7_ (biotin), and vitamin B_12_ (cobalamin) (24, 69, 79, 83, 89). As animals are unable to synthesize these essential vitamins, microbially mediated synthesis likely places a selective advantage on their maintenance, thereby contributing to the stability of the holobiont.

In summary, much of the understanding of metabolic interactions is derived from sequencing-based studies; however, some isotope-labeling experiments have revealed that symbiont-derived carbon and nitrogen can be transferred to sponge cells (62, 64, 80, 90) and that biomass transfer can also occur from SRB in at least one sponge species (85). Interestingly, nutrient transfer from symbionts to a host was recently found to be influenced primarily by host-symbiont identities and the ratio of gross productivity to respiration, rather than the actual symbiont abundance (90). Recent metaproteomic analyses have also revealed the expression of functions indicative of metabolic interactions between the host and symbionts, including transport functions for typical sponge metabolites (79). However, despite these insights, the highly complex sponge microbiome is likely to contain many more undescribed interactions, which need to be studied using both “-omics” and experimental research tools to fully understand the function of the sponge holobiont.

## MOLECULAR DETERMINANTS OF HOST-SYMBIONT INTERACTIONS

In a recent review, Bordenstein and Theis (91) argued that if microbial symbionts were faithfully transmitted across holobiont generations (either via vertical or horizontal transmission [see above]), then the following principles would apply: (i) the offspring’s microbiome would be more similar to that of the parent at a similar age than to that of unrelated adults in the population, (ii) experimentally tagged microorganisms from the adults would appear in their offspring more often than in the offspring of other species, and (iii) host immune systems, morphological structures, and/or behavioral mechanisms would be in place to promote the effective transmission and maintenance of beneficial microorganisms from parents to offspring (91). While our understanding of host specificity in sponge symbiosis largely substantiates the first of these principles (see above), little progress has yet been made to validate the second point. However, recent information has provided new insight into mechanisms that could allow for the recognition and maintenance of specific symbionts within the sponge.

Feeding studies have certainly confirmed that sponges are able to differentiate between food bacteria that enter the sponge and their own symbionts (92, 93), but whether this discrimination involves the host innate immune system or whether the symbionts themselves employ mechanisms to mask themselves from recognition and digestion is a topic of current research. Early studies suggested that sponge symbionts may produce physical barriers, such as mucous sheathes or capsules, to evade host detection (94). Recent research, however, has uncovered other potential molecular mechanisms of sponge-symbiont interactions, including symbiont proteins that contain eukaryotic domains (95). These domains found in the eukaryote-like proteins (ELPs) mediate protein-protein interactions for many biological processes, including processes involved in establishing an intracellular lifestyle. Interestingly, (meta)genomic analyses have shown that sponge microbial symbionts contain an abundance of genes encoding ELPs, such as ankyrin repeat proteins (ARPs), tetratricopeptide repeat proteins (TPRs), and leucine-rich repeat proteins (LRRs) (24, 69, 89, 96–98), and metaproteogenomic studies have confirmed that these proteins can be actively expressed (79, 99). In particular, the enrichment of ARPs in sponge symbiont genomes can be >5 times higher than what has been observed in other microbial symbionts and >20 times higher than in genomes of free-living marine bacteria (24). While the ankyrin repeat motif is a common protein-protein interaction motif thought to be involved in modulating cellular pathways needed for evolution of multicellular organisms, ARPs are also known to be secreted by bacterial pathogens, where they facilitate host infection and bacterial intracellular survival (100–103). To test whether these ARPs offered a mechanism to enable sponge symbionts to escape phagocytosis from the sponge, Nguyen and colleagues (95) used recombinant approaches to demonstrate that ARPs derived from sponge symbionts can modulate amoebal phagocytosis and impair phagosome development. These findings suggest that sponge amoebocytes may not actively “distinguish” between food bacteria and symbionts; rather, symbionts may contain specific proteins that allow them to manipulate host behavior. While these findings still need to be validated in native symbiont populations, they so far indicate that sponge symbionts may use these ELP-based molecular mechanisms for mediating host interactions.

Another potential molecular mechanism of host-symbiont interactions is quorum sensing (QS), which allows bacteria to sense and perceive their population density through the use of diffusible signals (104, 105). QS is known to be critically important in regulating some microbial symbioses (106), but its role in regulating host colonization and symbiont-symbiont interactions in sponges is relatively unexplored. The presence of the QS signal *N*-acyl homoserine lactone (AHL) has been frequently reported in a wide diversity of sponge species (107–109), but the importance of these compounds for holobiont interactions is still uncertain. In the most detailed analysis of AHL quorum sensing in sponges to date, Zan and colleagues identified genes for QS response regulators and signal synthases in a sponge-derived *Roseobacter* clade and provided evidence that AHL signaling contributes to flagellar motility, which may enable the symbionts to occupy different niches within the sponge environment (109).

Insights into how the host innate immune system may contribute to holobiont interactions have been afforded by the availability of a nearly complete genome for the sponge *Amphimedon queenslandica* (110). The genome-encoded pattern recognition receptors (PRRs), which are proteins expressed by cells of the host’s innate immune system to identify microbe-associated molecular patterns (MAMPS), pathogen-associated molecular patterns (PAMPS), or endogenous-damage-associated molecular patterns (DAMPS) (111), and may be used to recognize microbial ligands encountered within the holobiont. The binding of a PRR to a microbial ligand can set off a signal transduction cascade that can result in transcription of immune response genes encoding products such as antibacterial proteins (112). Interestingly, while the *A. queenslandica* genome does not encode Toll-like receptors (TLRs) or interleukin receptors (ILRs), which are among the best-characterized groups of PRRs, it does have two related receptors, which contain at least three extracellular interleukin 1 receptor-like domains and one Toll-interleukin 1 receptor domain (110, 113). However, none of these receptors contain the typical MAMP binding site, as they lack characteristic LRRs, and so it may be that the sponge receptors instead use extracellular immunoglobulin domains to interact with microorganisms (114), although this possibility still requires experimental validation (110). An alternative scenario is that lipopolysaccharide (LPS) binding-like proteins in sponges interact with other receptors to form a functional microbial sensor. A similar process occurs in *Hydra magnipapillata*, where a TIR (Toll-interleukin receptor) domain-containing protein lacking LRRs interacts with an LRR-containing protein to mediate the induction of antimicrobial peptides in response to PAMPs (115).

In *A. queenslandica*, a number of putative nucleotide oligomerization domain (NOD)-like receptor (NLR)-encoding genes that contain a NACHT domain in combination with LRRs have been detected (110). NLRs are highly diverse and in other organisms play a significant role in sensing a wide suite of different microorganisms (116–118). Most NLRs are situated in the cytosol, where they can directly respond to invading bacteria. Some NLRs can also be activated by extracellular bacteria, although the mechanisms that enable the receptor and bacterial ligand to interact in these instances are still unclear. Some evidence in other model systems suggest that NLR activators may be transported into host cells via bacterial type IV secretion systems and pore-forming toxins, and interestingly, type IV secretion systems have been found in sponge symbionts (24). In higher animals, NLRs detect peptides generated during the degradation of bacterial peptidoglycan (116), which enables effective discrimination between Gram-negative and Gram-positive microorganisms. NLRs can also detect endogenous DAMPs that are released from the host during cellular damage, which can stimulate the recruitment of immune cells to the damaged area (116). The predicted NLRs in the *A. queenslandica* genome may have the ability to detect both MAMPs and DAMPs, which may help the sponge host distinguish between pathogenic and symbiotic microorganisms (119), although further experimentation is required to support their role within the holobiont (9).

Scavenger receptor cysteine-rich (SRCR) proteins may also be involved in mediating sponge-microbe interactions. For instance, in vertebrates, macrophage class A scavenger receptor (SR-A) proteins mediate the endocytosis of bacteria, environmental particles, and DNA (120), and in humans, these proteins can bind to the LPSs of different bacterial species, resulting in phagocytosis (121). SR-A proteins have now been identified in multiple sponge species (110, 122, 123), and expression has been observed to increase in response to the presence of cyanobacterial symbionts (124). The large diversity of multiligand and multifunctional receptors generated by the SRCR protein superfamily is consistent with the immune requirements of a highly complex sponge holobiont, and further study of these receptors is expected to yield insights into the interaction dynamics within the hologenome.

## ENVIRONMENTAL ADAPTATION OF THE SPONGE HOLOGENOME

Within the sponge environment, both the host and symbionts need to cope with holobiont-generated stressors. For instance, symbionts need to survive the diverse array of host-generated natural products (reviewed in reference 9), including potent antimicrobial compounds, while the host must withstand potentially toxic conditions as a result of symbiont physiology, such as sulfide generation by SRBs (see above). Our current understanding of these coping mechanisms is superficial, although there is support for sponge symbionts acquiring resistance mechanisms specifically tailored to their stressful host environment. Genomic analysis of sponge symbionts (24, 70, 97) has shown that symbionts carry universal stress proteins, which are known to respond to a wide variety of stressors, including nutrient starvation, heat exposure, acid exposure, heavy metal exposure, oxidative stress, osmotic stress, and antibiotics.

As active filter feeders with few external barriers, both host and symbionts also need to cope with the local conditions specific to their environment. Many studies have assessed changes in the compositions of sponge microbiomes in response to temperature (78, 99, 125–129), pH (130), nutrients (131, 132), contaminants (133–135), and sediment loads (136, 137). Some of these studies found correlations in microbial shifts, with declining host health, while others revealed remarkably stable microbial communities irrespective of environment and/or host health state. However, few studies have dissected cause-effect pathways for environmental-stress-related dysbiosis or explored how environmental factors affect the hologenome. A notable exception was a temporal study in which expression profiling of the sponge host was performed in conjunction with phylogenetic, functional, and expression analysis of the symbiont community in response to elevated temperature (99). Prolonged thermal stress was found to change the sponge-associated microbial community from one with predominantly symbiotic functions to one characterized by opportunistic bacterial functions. Importantly, host gene expression and metaproteomic analyses revealed that elevated temperature precipitated an immediate stress response in both the host and the symbiont community, including a reduction in expression of functions that mediate the symbiotic partnership. For instance, disruption to nutritional interdependence and molecular interactions (such as reduced expression of transporters involved in the uptake of sugars, peptides, and other substrates) occurs in the early stages of heat stress, and this likely destabilizes the holobiont, ultimately leading to the loss of typical sponge symbionts and the introduction of new microorganisms with functional and expression profiles indicative of a scavenging lifestyle (e.g., a lack of virulence functions and high growth rates) (99).

The interactions occurring within the sponge hologenome during disease are even less explored than during environmental perturbation. Reports of sponge disease span a wide range of species (reviewed in references 138 to 145) and biogeographies (reviewed in reference 138), and while some disease events correlate with prevailing environmental conditions and many correlate with a shift in the host-associated microbiome, there are almost no confirmed etiological agents and no understanding of the molecular interactions occurring within the holobiont during infection or disease progression. The one exception is a disease affecting the Great Barrier Reef sponge *Rhopaloeides odorabile* (146, 147), in which the pathogen was found to contain virulence genes related to a collagenase activity that was the primary cause of pathogenicity (148). The failure to identify specific causative agents suggest that diseases or disease-like syndromes may be a result of complex functional interactions occurring within the holobiont, and future experimental research is required to generate a better understanding of the links between host health, symbionts, pathogens, and the environment.

When considering the adaptive capacity of sponge holobionts to environmental change, the microbiome offers significant and mostly unrecognized potential (91). While the evolutionary theory defined by Lamarck as inheritance of acquired traits was largely displaced by Darwinism (149), environmentally acquired fluctuations in the microbiome can have significant functional consequences for the holobiont phenotype (as outlined above), upon which selection may act. For instance, environmentally induced changes in microbial abundance within the sponge holobiont may be akin to gene duplication events, and community shifts as a result of environmental change can either remove or introduce raw genetic material into the hologenome, upon which selection may act. Symbiont transmission is a key feature in maintaining the sponge holobiont, which would enable these environmentally acquired traits to be successfully passed to offspring, ultimately resulting in transgenerational adaptation or speciation of the sponge hologenome.

## FUNCTIONAL CONVERGENCE AND EVOLUTION OF THE SPONGE HOLOGENOME

As recently raised by Moran and Sloan (61), for coevolution to occur in a holobiont context, evolutionary change must be driven by selective forces enacted by the other holobiont members. Intimate associations that affect host fitness do not necessarily require a history of coevolution, and host-associated microorganisms can have mutualistic partnerships without necessarily having undergone natural selection to benefit their hosts. Although selection at the level of the symbiotic community or sponge hologenome may occur in some cases, it is certainly not an exclusive or catch-all mechanism to explain all features of sponge-symbiont associations, and considerable research is still required to tease apart the evolutionary history of sponge microbial symbioses. The extreme diversity of sponge holobionts, often including >100 species/strains of a single symbiont lineage within a single sponge species (27), is particularly perplexing and raises questions about (i) the level of niche differentiation occurring within the sponge holobiont, (ii) the level of functional redundancy within symbiont communities, (iii) the homeostatic mechanisms that support such diverse yet specific associations, and (iv) the holobiont traits on which selection may act to maintain such genetic diversity.

The substructure of the sponge (pores, channels, choanocyte chambers, pinacoderm, etc.) likely provide distinct microenvironments that support different types of symbionts (see, e.g., reference 150), thus allowing niche differentiation. However, most previous work has analyzed symbiont communities in bulk tissue, and there is a greater need to define spatial differentiation of function as a potential explanation for the large symbiont diversity observed in sponges. An alternative explanation for the large number of related strains may lie in the absence of strong positive selections that would result in selective sweeps. In fact, recent analyses of sponge genomes indicate a large degree of population heterogeneity and an abundance of mobile genetic elements (97, 98) that could drive genetic diversification under relaxed selective pressure.

Until recently, it was also unknown whether, or to what extent, the diverse communities of microbial symbionts occupying equivalent niches in different sponge species shared functions and, if so, whether these equivalent functions were undertaken by evolutionarily convergent mechanisms. Research employing isotope analysis has shown that sponges that appear to exploit the same resources may in fact occupy different niches with respect to nitrogen and carbon metabolism (151). Analysis of isotope ratios from a range of sponge species spanning a gradient of photosymbiont abundance found that host identity explained most of the variation in isotope values, although photosymbiont abundance also explained a significant proportion of the variation in some species (151). This between-species variation indicates that species-specific microbiomes likely enable sponge species to exploit different nutrients within a particular reef system.

To further address the concept of functional equivalence, Fan and colleagues used metagenomic sequencing to compare the phylogenetic and functional microbiomes of six sponge species. Despite large differences in the taxonomic compositions of the microbiomes, shared core functions were still identified across phylogenetically disparate host species (24). These core functions were consistent with the described biological and ecological roles of sponge symbionts, such as nitrogen metabolism and nutrient utilization (see above). Importantly, these core functions were found to be provided by analogous enzymes and biosynthetic pathways, with symbiont communities in divergent hosts appearing to have evolved different genomic solutions to perform the same function or to occupy the same niche (24). For example, different sponges were found to host distinct microorganisms that use different enzymes (e.g., NapA versus NarG or NirK versus NirS) to perform equivalent functions in denitrification, although why the preference for respiratory nitrate and nitrite reductases, respectively, is still unclear. Further evidence of functional convergence in sponge symbiosis was provided by analyzing the microbiome compositions of several temperate sponge species in conjunction with an analysis of the uptake and release of dissolved organic carbon (DOC), dissolved organic nitrogen (DON), dissolved inorganic nitrogen (DIN), and phosphate, revealing that different metabolic pathways were mediated by unique symbiont communities in each host species (152). Similarly, GeoChip analysis performed in conjunction with microbiome profiling has revealed considerable equivalence in the functional gene repertoires of sponges, including between HMA and LMA species (see above).

**FIG 2:**
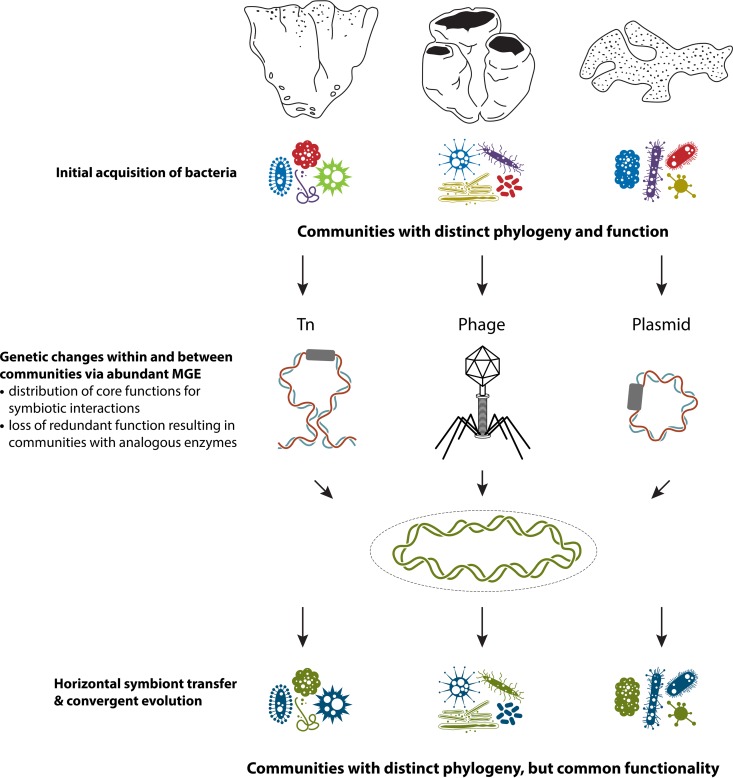
Working model of how sponge symbiosis has evolved toward microbial communities with distinct phylogeny but common functionality. Differently shaped bacteria represent distinct taxa, while different colors represent functions. As genetic exchange occurs within and between symbiont communities, their functions converge.

One explanation for this functional convergence lies in the evolutionary processes that shape communities. Both niche selection and neutral hypothesis models are used to model the community structure of microbial communities (153–155). The initial host acquisition of symbionts, the capacity for the symbiont to be vertically transmitted, and the evolution of an obligate relationship are likely to be primary determinants in how these different processes structure community assembly in sponge holobionts (7). Conceivably, different types of free-living microorganisms are likely to have initially entered into low-selectivity or random (i.e., neutral) associations, and hence different sponge species would have acquired different phylogenetic clades of microorganisms, leading to the species-specific taxonomic profiles seen in many sponge studies (see above). However, as the symbiotic relationship evolves and vertical transmission occurs (26, 156), symbionts maintain or acquire functions that stabilize their interaction with their host. Thus, for different host species with similar functional niches, symbionts will eventually converge functionally (Fig. 2).

Random nucleotide changes, recombination events within and between chromosomes, gene duplications and/or losses, and horizontal gene transfer (HGT) within and between members of the holobiont all offer mechanisms that could produce genetic variation that underpins sponge hologenome evolution. Beneficial, deleterious, and neutral mutations can occur in any part of the sponge holobiont, but the short generation time of microorganisms compared to that of the host might represent a more important source of variation. These modifications can lead to phenotypic variation upon which both natural selection and genetic drift may operate. The evolutionary adaptation of microbial populations to specific niches is largely facilitated through mobile genetic elements (MGEs), including transposons, plasmids, and prophages (157). In comparison to free-living microorganisms, sponge symbionts appear to be enriched in HGT systems, including transposases, conjugative transfer systems, and retro elements containing reverse transcriptases and integrases as well as genetic systems for transformation (24, 97). Each sponge species also tends to have its own unique set of HGT systems to facilitate this genetic exchange. For instance, while genes for transposases are generally abundant in sponge symbiont communities, the specific types of transposase genes are unique to each host species, with no evidence of biogeography. This suggests limited dispersal of mobile genetic elements between sponge species (24) and instead argues that the MGEs are more important for within-community adaptation to a specific host.

Another important agent of genetic variation is viruses, which have been suggested to be critical to holobiont function and hologenome adaptation in other host systems (158). To date though, almost nothing is known about their role in sponges (159). Phage-mediated transduction can cause lysis and death of bacterial cells (160), and considering the large amount of viruses present in seawater (10^7^ /ml), sponges are likely to encounter a large diversity of viruses through their filtering activities. It appears therefore necessary that sponge-associated microorganisms have very effective mechanisms to prevent phage infection or lysis and to control excessive transduction to minimize the introduction of foreign DNA into the hologenome. Excessive HGT from external sources can erode genomic integrity (161), and there is genomic evidence that sponge holobionts might control this by abundant restriction-modification (R-M) systems and toxin-antitoxin (T-A) systems, as well as clustered, regularly interspaced, short, palindromic repeats (CRISPRs) and CRISPR-associated (CAS) proteins (24). The heritable and adaptive CRISPR/CAS system confers resistance to foreign genetic material by acquiring short DNA sequences from the invading phage or plasmid and incorporating them into an array of spacer sequences that can be hybridized to invading DNA, which is subsequently degraded by CAS proteins (162). There appears to be very little overlap in repeats and spacers in the microbiomes of different sponge species (24), which is consistent with the notion that different host species harbor distinct microbial communities that experience attacks by distinct viral populations. An enhanced understanding of the viruses inhabiting the sponge holobiont and the role of viruses as mediators of genetic transfer are areas of current research that will undoubtedly reveal unique insights into the sponge hologenome in coming years.

The highly abundant and diverse MGEs described above may contribute to evolutionary processes in the hologenome in a number of ways. First, MGEs can mediate HGT and distribute essential core functions among community members that facilitate the evolutionary adaptation of specific symbionts to the host environment (163). As a consequence, individual genomes from different phylogenetic lineages should become more similar to each other, such as with the pattern reported for mammalian gut bacteria (164) (Fig. 2). Second, adaptation to the host environment can result in the removal of nonessential genes (165), such as with the likely loss of the photolyase or methionine synthesis genes reported for sponge symbionts (24), and this process can be mediated by increased transposon density (166), a pattern also characteristic of sponge symbiont metagenomes (24). Third, individual symbiont genomes may use MGEs to eliminate functions that are already provided by genomes of other community members, which would ultimately lead to niche specialization, such as occurs during nutritional interdependence (167). Within the sponge hologenome, different genes would be available for different tasks, consistent with findings of functional equivalence (see above). However, teasing apart these fine-scale interactions is challenging, and future studies that combine genomic and metaproteogenomic approaches with physiological experiments will provide unique insights into sponge symbiosis and the factors driving hologenome evolution.

Embracing the hologenome concept also means that there should be no barriers for HGT between the host and the symbiont lineages. However, specific examples of HGT in sponges are rare, likely due to the dearth of genomic data available for both sponges and their symbionts. One outstanding example is the likely HGT of a spherulin-encoding gene from a bacterial lineage to a sponge host (168). Spherulin is most likely involved in skeletogenesis, which suggests that this HGT event may have contributed to the evolution of the sponge body plan. Another example is the likely transfer of ELPs from the sponge host to the symbiont, which may play a role in regulating phagocytosis (95; see above). Finally, the mitochondrial genome of the sponge *Tetilla* sp. was found to contain introns that appear to have been transferred from a fungal host (169), although the functional significance of this HGT event is still unclear. Taken together, these findings indicate that HGT between host and symbionts (and vice versa) does indeed occur, and careful examination of existing and emerging genomic data on the sponge hologenome will likely reveal many more examples. HGT transfer between the host and symbionts is expected to increase the interdependency of the symbiosis, as a given essential function might be maintained only in one genome and lost in all other holobiont members. This would further drive holobiont relationships from facultative toward obligate. The fact that many sponge symbionts cannot be cultured outside their host and that removal of symbionts (e.g., via antibiotic treatments) has proven challenging to achieve without deleterious outcomes for the sponge, indicates that the sponge hologenome has undergone an evolutionary progression toward such an obligate symbiosis.

## CONCLUSIONS

This review provides a synthesis of knowledge related to the sponge holobiont and its hologenome, with a particular focus on the molecular and physiological determinants of holobiont interactions using insights obtained with “-omic” data and hypothesis-driven experimental research. However, despite significant recent progress in our understanding of the sponge holobiont, large-scale community sequencing initiatives targeting both the host and the microbiome would further illuminate the complex network of holobiont interactions, including shared metabolic pathways, the potential role of the microbiome in environmental adaptation of the holobiont, and the genomic interplay occurring between the various components of the holobiont.
